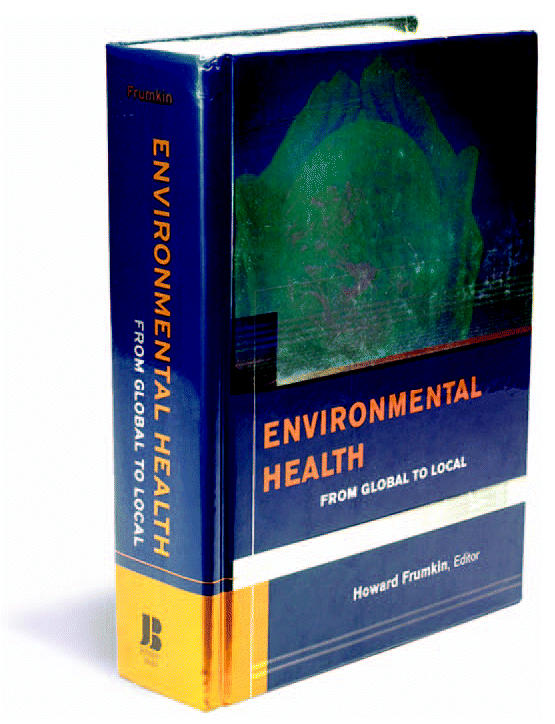# Environmental Health: From Global to Local

**Published:** 2006-11

**Authors:** Bernard D. Goldstein

**Affiliations:** Bernard D. Goldstein is professor of environmental and occupational health and former dean at the University of Pittsburgh Graduate School of Public Health. He is formerly U.S. Environmental Protection Agency Assistant Administrator for Research and Development and served as president of the Society for Risk Analysis. He is a member of the Institute of Medicine, where he serves on the Board of Health Sciences Policy and on its Environmental Health Roundtable

Edited by Howard Frumkin

San Francisco:Jossey-Bass, 2005. 1,108 pp. ISBN: 0-7879-7383-1, $75.00

Howard Frumkin has been a leader in expanding the definition of environmental health beyond the effects of toxic chemicals. His eloquence and breadth of understanding are evident in the introductory chapter to this textbook, as well as in his chapter “Nature Contact: a Health Benefit?” The book’s 36 chapters contain highly pertinent insights and information on environmental issues that go beyond the usual boundaries of classic environmental health. Among the many excellent chapters are ones on climate change, ecology, urbanization, environmental justice, developing nations, health care services, energy production, genetics, indoor air pollution, religious issues, clinical services, legal remedies, environmental health policy, and transportation. This breadth makes the book a very useful reference source. Unfortunately, so much of the basics of classic environmental health are omitted or insufficiently presented that the book is not suitable as a textbook for standard undergraduate or graduate environmental health courses.

Core concepts in classic toxicology and in risk assessment related to environmental chemicals receive minimal attention. For example, there is little or nothing on such topics as threshold and nonthreshold dose responses; traditional chemical safety factors; reference doses; weight of evidence for carcinogenicity; and other standard approaches to evaluating personal and community risk from chemicals. The linkage between environmental exposure and dose, including internal dose and dose to target tissue, is only scantily presented. Classic environmental health concepts such as bioavailability, bioaccumulation, and biomagnification are not systematically addressed. The risk assessment chapter focuses almost totally on cancer risk, but without mentioning the International Agency for Research on Cancer or the National Toxicology Program processes for hazard identification of carcinogens. Cumulative risk is briefly touched on in the excellent chapter on environmental justice—but with no mention of aggregate risk. Environmental indicators are discussed only in relation to water pollution, and the exciting new advances in this area are not integrated or referenced; biomonitoring is only briefly mentioned in the discussion of industrial hygiene; and biomarkers only in relation to exposure, but not to effect or susceptibility. These basic concepts, as well as information about the health effects and mechanisms of toxicity of major environmental chemicals, are central to teaching the environmental health sciences to undergraduate and graduate students.

Emblematic of the disconnect between the excellence of a chapter and value as a textbook is the treatment of exposure assessment and of informatics. The superb chapter on industrial hygiene only peripherally addresses environmental exposure assessment, a crucial component of classic environmental health. Omitted are conceptual and technical advances that have contributed heavily to advances in environmental protection. This chapter does contain a brief discussion of dose issues missing from the toxicology chapter, although its example of carbon monoxide is unfortunate in omitting time to equilibrium and CO production through normal metabolism. Similarly, informatics has developed two major areas in environmental health: geographic information systems (GIS) and computational chemical toxicology. GIS receives a full and informative chapter, but the impact of informatics on computational toxicology is barely mentioned.

The coverage of toxicity and health effects is excellently provided for radiation and for pesticides. For each major type of radiation energy, Arthur C. Upton discusses sources, mechanisms of action, acute and chronic effects, assessment of exposure and risk, effects in susceptible populations, and prevention and mitigation. Similarly, an excellent chapter on pesticides describes pests and their impact on human health, classifies pesticides by target and chemical structure, discusses pesticide use and exposures and the regulation of pesticides, and describes integrated pest management. But there is nothing like this for the chemical agents that remain classic areas of concern for environmental health.

Some missing chemicals and concepts are covered in passing in other chapters. Unfortunately, the index is very poorly done. For effective use as a textbook, the index must be a means for the student to pursue specific topics. Yet major items are unaccountably left out. As examples, despite four different references to benzene among the chapters, benzene is not listed in the index; CO is indexed as a criteria air pollutant, but not to the other two chapters in which it is discussed; Frumkin’s introductory discussion of dioxins does not lead to a listing in the index; and the only index listing for lung cancer is to a page that devotes twice the space to leukemia—but leukemia is not indexed.

The authors are to be commended for using a fact-based approach, although there is occasional preaching. Joel A. Tickner, a lucid advocate of the precautionary principle, does not address its track record of being misused for economic advantage, and erroneously asserts that “strong” epidemiologic evidence of risk is needed for preventive approaches based upon risk assessment. We can agree with Barry S. Levy and Victor W. Sidel that public health personnel should advocate action against the evils of landmines without being told that we have a “responsibility” to advocate for a specific treaty.

Among the superb chapters in this book are those by Sarah Kotchian on the practice of environmental public health and by Vincent T. Covello on the communication of environmental health risk. But students not only must learn who should communicate, and how, but also understand the basics about chemicals and their effects. This would be expected in a textbook for a course adhering to the recent core competency project of the Association of Schools of Public Health (http://www.asph.org/UserFiles/FinalVersion2.1.pdf). Perhaps revision for the next edition of this otherwise superb reference work will make it suitable as a textbook for undergraduate and graduate courses in environmental health.

## Figures and Tables

**Figure:**